# Differential expression of tear film cytokines in Stevens–Johnson syndrome patients and comparative review of literature

**DOI:** 10.1038/s41598-021-97575-y

**Published:** 2021-09-16

**Authors:** Madhuri Amulya Koduri, Deeksha Prasad, Shriya Upadhyaya, Jilu Jaffet, Swapna S. Shanbhag, Sayan Basu, Vivek Singh

**Affiliations:** 1 Prof. Brien Holden Eye Research Institute (BHERC), L V Prasad Eye Institute, Road No.2, Banjara Hills, Hyderabad, 500034 Telangana India; 2 Manipal Academy of Higher Education (MAHE), Manipal, Karnataka India; 3 Center for Ocular Regeneration (CORE), L V Prasad Eye Institute, Hyderabad, Telangana India

**Keywords:** Chemokines, Cytokines, Immunological disorders, Biomarkers, Corneal diseases, Eyelid diseases

## Abstract

To investigate the differential expression of tear cytokine levels among chronic Stevens–Johnson syndrome (SJS) patients to better understand the role of significantly altered cytokines in disease development. Tear samples were collected using Schirmer strips in 24 eyes of chronic SJS, 24 eyes of age and gender-matched controls, and 14 eyes of aqueous deficiency dry eye disease (DED) patients. The cytokine analysis was performed among 18 analytes which include pro-inflammatory, anti-inflammatory factors, and ELR-negative CXC chemokines. String analysis was performed for the significantly altered cytokines to understand their co-expression and role in the disease development. Additionally, a literature review was conducted to identify the signature cytokines present in chronic SJS tears. The differential expression of IL-6 (p ≤ 0.029), CXCL8/IL-8 (p ≤ 0.009), IL-1β (p ≤ 0.041), IL-2 (p ≤ 0.025), IL-10 (p ≤ 0.053), and CXCL-10 (p ≤ 0.044) were observed in chronic SJS patients and healthy controls. Whereas, IL-6 (p ≤ 0.029), CXCL8/IL-8 (p ≤ 0.058), CCL4 (p ≤ 0.056), GM-CSF (p ≤ 0.0001) IL-10 (p ≤ 0.025), and CXCL-10 (p ≤ 0.010), were differentially expressed in SJS as compared to severe DED patients. String analysis of the significantly altered cytokines revealed the involvement of several biological processes including the chronic inflammatory response, nitric oxide synthesis, angiogenesis, and cellular response to drugs. Among all the cytokines evaluated, the expression of CXCL8/IL-8 and CXCL10 levels were consistently reported in the literature. There was a differential expression of tear cytokines in SJS when compared to DED and healthy controls. The differential expression of CXCL8/IL-8 and CXCL10 was in line with existing literature and their role in chronic SJS pathogenesis merits further evaluation.

## Introduction

Stevens–Johnson syndrome/toxic epidermal necrolysis (SJS/TEN) is a rare, acute, and serious, mucocutaneous drug reaction clinically characterised by blister formation and epithelial sloughing in the acute stage^1^. Even though the incidence of the disease ranges from 0.6 to 12 cases per million population per year in various populations, the severity of the disease can be extreme and therefore demands utmost and urgent care^2^. One of the well-known triggers of SJS is drug induced hypersensitivity whereas others are bacterial and viral infections^3^. According to a systematic review conducted in India, the most common drugs responsible for SJS were sulphonamides (37%), anti-epileptics (36%), and NSAIDS (16%) with an overall mortality of 13%. In a nine-year study, 19.5% of hospitalized patients with severe cutaneous adverse reactions in India were attributed to SJS. Therefore, it is widely speculated that the incidence of SJS could be higher in India as compared to the Western hemisphere^4,5^.

All the mucosal surfaces including ocular, genital, gastro-intestinal, nasal, renal, pulmonary, and buccal cavity can be involved at the disease onset^6,7^. However, the chronic sequelae of SJS are frequently ocular and include eyelid margin keratinization, meibomian gland dysfunction, conjunctival cicatrization and progressive corneal damage, which can be potentially blinding. Ocular surface dryness is another important consequence in SJS patients where the combined involvement of aqueous tear deficiency, decreased ocular surface wettability and an elevated rates of tear evaporation are observed^8,9^. At the cellular level there are distinct immunological responses occurring in SJS, which involves the secretion of cytokines, co-stimulatory and inflammatory molecules^10^. Hence, recent studies in the field of ocular inflammatory diseases have been focused on cytokine profiling of ocular fluids like tears and vitreous. Cytokine studies aid in the diagnosis of the disease and also for specific therapeutic management of the disease condition which are often associated with inflammation. For example, identification of these cytokines levels makes the classification of ocular allergic diseases possible^11^.

In the present study, we designed a customized panel to analyse the tears from chronic SJS patients for the expression of pro-inflammatory cytokines IL-1β, IL-2, IL-6, CXCL8/IL-8, IL-15,IL-17A, bFGF, RANTES, MCP-1, GM-CSF, TNF-α, IFN-γ and anti-inflammatory cytokines IL-10, IL-13 along with ELR-negative CXC chemokines CXCL9 and CXCL10. We explored and analysed the tears of Indian patients reporting to a tertiary eye centre and looked for differential expression of our designed panel of cytokines and compared with the healthy controls and severe dry eye disease (DED) patients. We also performed String analysis which helped to explore various plausible biological processes that could be linked with altered cytokines in chronic SJS tear samples. We believe our study findings will be helpful in better understanding of the SJS disease process in the eye and lead to insights that may eventually lead to better management and improvement in treatment outcomes.

## Results

**Figure 1:**
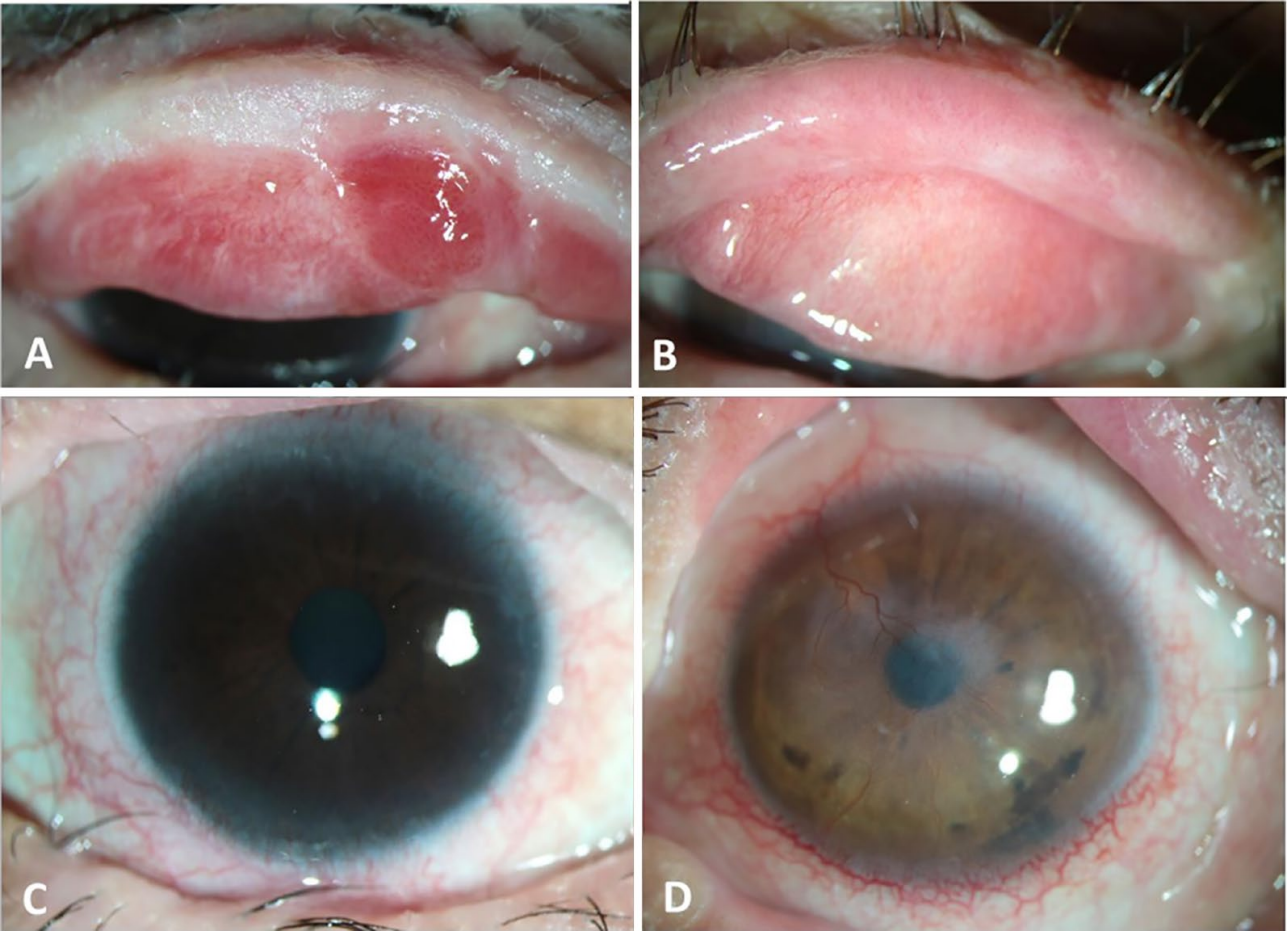
Ocular characteristics in eyes with chronic Stevens–Johnson syndrome. (**A**) Lid margin keratinization with tarsal conjunctival scarring in the upper eyelid (patient 11); (**B**) Upper lid post lid margin mucous membrane graft (patient 9); (**C**) peripheral corneal vascularization (patient 11); (**D**) Corneal scarring with vascularization with peripheral limbal vascularization (patient 9).

In this study, SJS patients (n = 12, 24 eyes; 7 males/5 females; mean age: 32.25 ± 12.12 years) (as shown in Fig. 1), healthy controls (n = 12, 24 eyes; 7 males/5 females; mean age: 30.8 ± 10.34 years) and non-SJS aqueous deficiency DED patients (n = 7, 14 eyes; 2 males/5 females; mean age: 43.5 ± 12.73 years) were recruited. The controls were age-matched/gender-matched volunteers and the tear collection time and total protein concentration in controls, SJS and DED are given in Table 1. Additionally patient details including age, gender, duration since onset of drug reaction, drug affected with and clinical symptoms examined under slit lamp microscope were detailed in Table 2. The data analysis with respect to the usage of topical steroids/anti-inflammatory drugs showed patients who are not under topical steroids have down-regulation of CXCL10 (p value ≤ 0.06) and IL-10 (p value ≤ 0.042) compared to patients who are on topical steroids/anti-inflammatory drugs. Simultaneously, the pro-inflammatory factors IL-2 (p value ≤ 0.04) and bFGF (p value ≤ 0.05) were upregulated in patients who are not on topical steroids/anti-inflammatory drugs (the bilateral usage of topical steroids and its dosage is given in the Supplementary Table S1). On further analysis of gender based effects in the SJS group, CXCL9 (p value ≤ 0.05) and IL-15 (p value ≤ 0.02) were significantly upregulated in females, otherwise none of the cytokines in the panel has shown any gender bias.

**Table 1:** The table below shows the age and gender of chronic SJS, healthy controls and severe DED patients. Also the schirmer value and total protein concentration of the collected tears. Also the schirmer value and total protein concentration of the collected tears.

Subjects	Age	Gender F:M	Schirmer value OD:OS	Schirmer time (in min)	Total protein concentration
Controls	30.8 ± 10.34	5:7	29.3 ± 1.48: 24.9 ± 7.52	3.92 ± 0.96	3252.6 ± 2444.55
SJS	32.25 ± 12.12	5:7	8.58 ± 4.52: 9.66 ± 3.86	5	1540.13 ± 972.97
Severe DED	43.5 ± 12.73	5:2	4.2 ± 0.76: 4 ± 0.81	5	1866.31 ± 858.57

**Table 2:** Clinical characteristics of patients involved in this study with the slit lamp examinations of each of a patient.

SJS Patients	Age (in years)	Gender	Duration since onset of disease	Drug sensitivity for	Significant ocular findings examined on slit lamp				COCS grade
						Eyelids and eyelid margin	Conjunctiva	Cornea	
Patient 1	26	F	11Y	Cefixime	RE	Few blocked orifices, thickened lid margins	Hyperemia	Scarring with 360 degree vascularisation	16
					LE	Few blocked orifices, thickened lid margins	Hyperemia	Scarring with 360 degree vascularisation	15
Patient 2	22	M	12Y	Paracetamol	RE	Thickened lid margins, symblepharon at medial canthus	Hyperemia	Superficial punctate keratopathy	6
					LE	Thickened lid margins, symblepharon at medial canthus	Hyperemia	Superficial punctate keratopathy	6
Patient 3	30	M	1Y/2 M	Sulphonamide	RE	Thickened lid margins, loss of eyelashes	Hyperemia, cicatrization	Vascularization with pannus	27
					LE	Thickened lid margins, loss of eyelashes	Hyperemia, cicatrization	Vascularization with pannus	26
Patient 4	34	M	7 M	antibiotics	RE	Lid margin mucous membrane graft	Normal	Superficial punctate keratopathy	11
					LE	Lid margin mucous membrane graft	Hyperemia	Superficial punctate keratopathy	13
Patient 5	23	F	10Y	Sulphonamide	RE	Lid margin keratinization	Normal	Vascularization	13
					LE	Lid margin keratinization	Normal	Vascularization	16
Patient 6	44	M	10Y	Sulphonamide	RE	Thickened lid margins, loss of eyelashes	Mild hyperemia	Scarring, superficial vascularization	13
					LE	Thickened lid margins, loss of eyelashes	Mild hyperemia	Scarring, superficial vascularization	13
Patient 7	60	M	12Y	Sulphonamide	RE	Lid margin keratinization	Hyperemia	Scarring, vascularization, conjunctivalisation	27
					LE	Lid margin keratinization	Hyperemia	Scarring, vascularization, conjunctivalisation	26
Patient 8	24	F	6Y	NA	RE	Lid margin keratinization	Superior symblepharon	Scarring, vascularization	34
					LE	Lid margin keratinization	Superior symblepharon	Scarring, vascularization	35
Patient 9	37	F	2Y	Penicillin	RE	Lid margin mucous membrane graft	Hyperemia	Scarring with vascualrization	11
					LE	Lid margin mucous membrane graft	Hyperemia	Scarring	23
Patient 10	19	M	2Y	Sulphonamide	RE	Distichiatic lashes	Hyperemia	Superficial corneal vascularization	17
					LE	Distichiatic lashes	Hyperemia	Superficial corneal vascularization	18
Patient 11	44	F	7Y	Cephalosporin	RE	Lid margin keratinization	Hyperemia	Superficial punctate keratopathy, peripheral corneal vascularization	15
					LE	Thickened eyelid margins, loss of eyelashes	Hyperemia	Superficial punctate keratopathy, peripheral corneal vascularization	13
Patient 12	24	M	5Y	NA		Lid margin mucous membrane graft	Hyperemia	Peripheral corneal vascularization	11
						Lid margin mucous membrane graft	Hyperemia	Peripheral corneal vascularization	11

In Gender, *F* female, *M* male; in duration since onset of disease, *Y* years, *M* months; Ocular complications examined on slit lamp, *RE* right eye and *LE* left eye, *COCS* chronic ocular surface complication score [Score as given in Sotozono et al.^26^].

### Pro-inflammatory cytokines in chronic SJS tears

**Figure 2:**
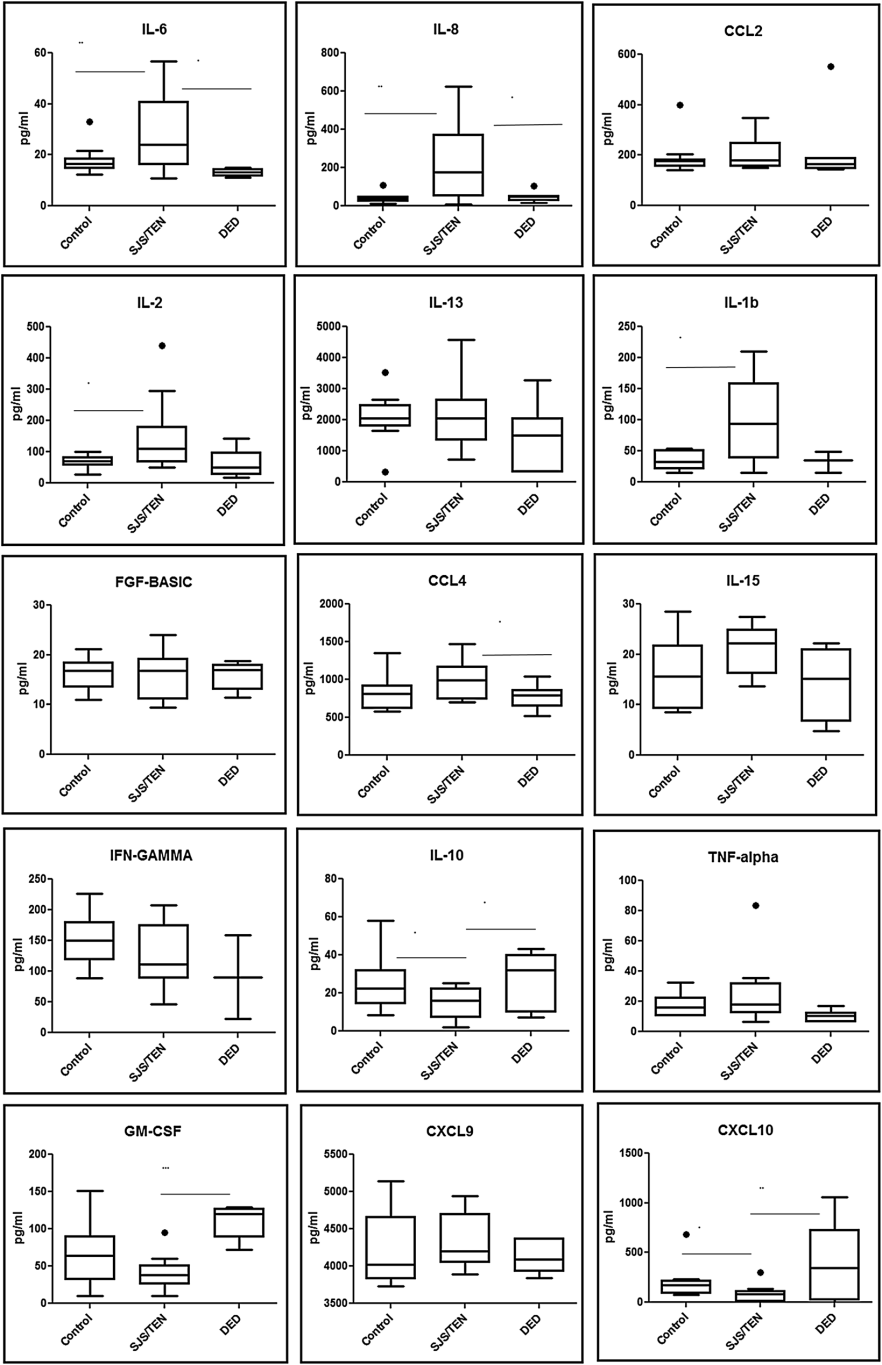
Above figure shows the cytokine analysis in tear samples using multiplex ELISA. The expression of cytokines in chronic SJS tears are compared with the controls and severe DED tears. *p value ≤ 0.05, **p value ≤ 0.01, ***p value ≤ 0.001.

We observed significant upregulation of pro-inflammatory cytokines IL-6 (p-value ≤ 0.029), CXCL8/IL-8 (p-value ≤ 0.009), IL-1β (p-value ≤ 0.041) and IL-2 (p-value ≤ 0.025) in tears of chronic SJS patients when compared to controls. There is no significant difference found in the levels of TNF-α, IFN-γ, CCL2, CCL5, CCL4, IL-17A, GM-CSF, FGF-basic, IL-15 and CCL11 in tears of control vs SJS patients. The comparative analysis of the tear samples in chronic SJS patients with DED patients revealed that IL-6 (p-value ≤ 0.015) was significantly upregulated, simultaneously, GM-CSF (p-value ≤ 0.0001) was highly downregulated (Fig. 2). There was no significant differential expression of CXCL8/IL-8, CCL4, TNF-α, CCL2, IL-1β, IL-17, IL-2, IFN-γ, IL-15, IL-17A, FGF-basic and CCL11 among severe DED and chronic SJS tears.

### Anti-inflammatory cytokines in chronic SJS tears

The anti-inflammatory cytokine IL-10 (p-value ≤ 0.053) was found to be downregulated but not statistically significant in Chronic SJS tears when compared with controls. The comparative analysis of IL-10 (p-value ≤ 0.025) in SJS tears with severe DED was significantly downregulated (Fig. 2) and IL-13 didn’t exhibit any significant difference in chronic SJS tears compared to controls and severe DED.

### ELR-negative CXC chemokines in chronic SJS tears

Among the two ELR-negative CXC chemokines in this study, CXCL10 was significantly downregulated when compared with healthy controls (p value ≤ 0.044). The CXCL10 levels in the SJS patients (p value ≤ 0.010) were significantly downregulated when compared with severe DED tears. Whereas, CXCL9 didn’t show any significant change in SJS patients in comparison with controls and severe DED tears (Fig. 2).

### String analysis

**Figure 3:**
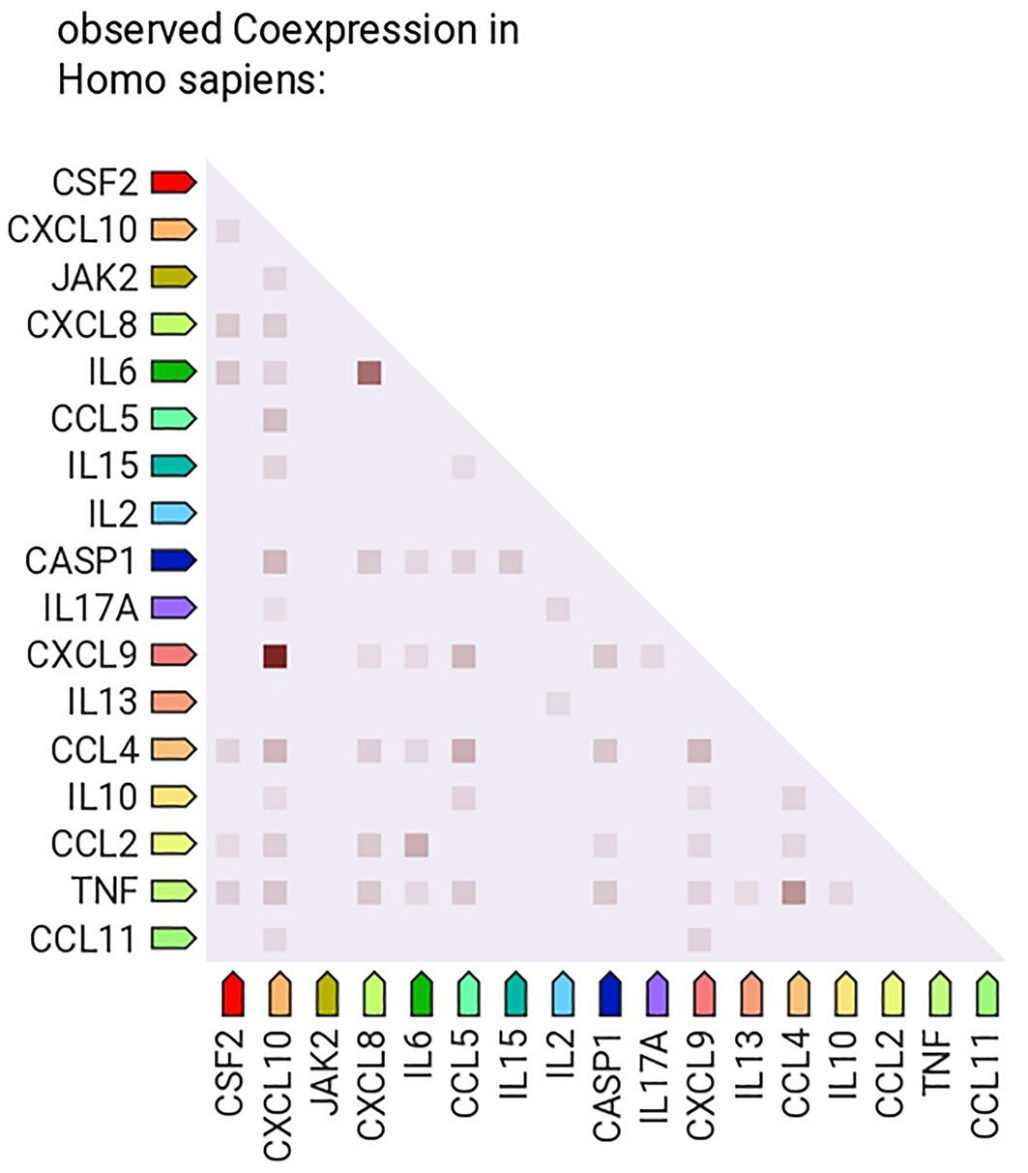
Above data was generated using String software analysis from the existing literature. The figure illustrates the co-expression of the cytokines in normal homeostatic conditions which are reported in this study, where it is found that CXCL10 and CXCL9 are highly co-expressive (RNA co-expression score 0.848). And the next highest co-expressive chemokines are CXCL8/IL-8 and IL-6 which are pro inflammatory cytokines (RNA co-expression score 0.581). The results obtained in the current study reveals that the co-expression of CXCL10 and CXCL9 was lost in chronic SJS patient tear.

**Figure 4:**
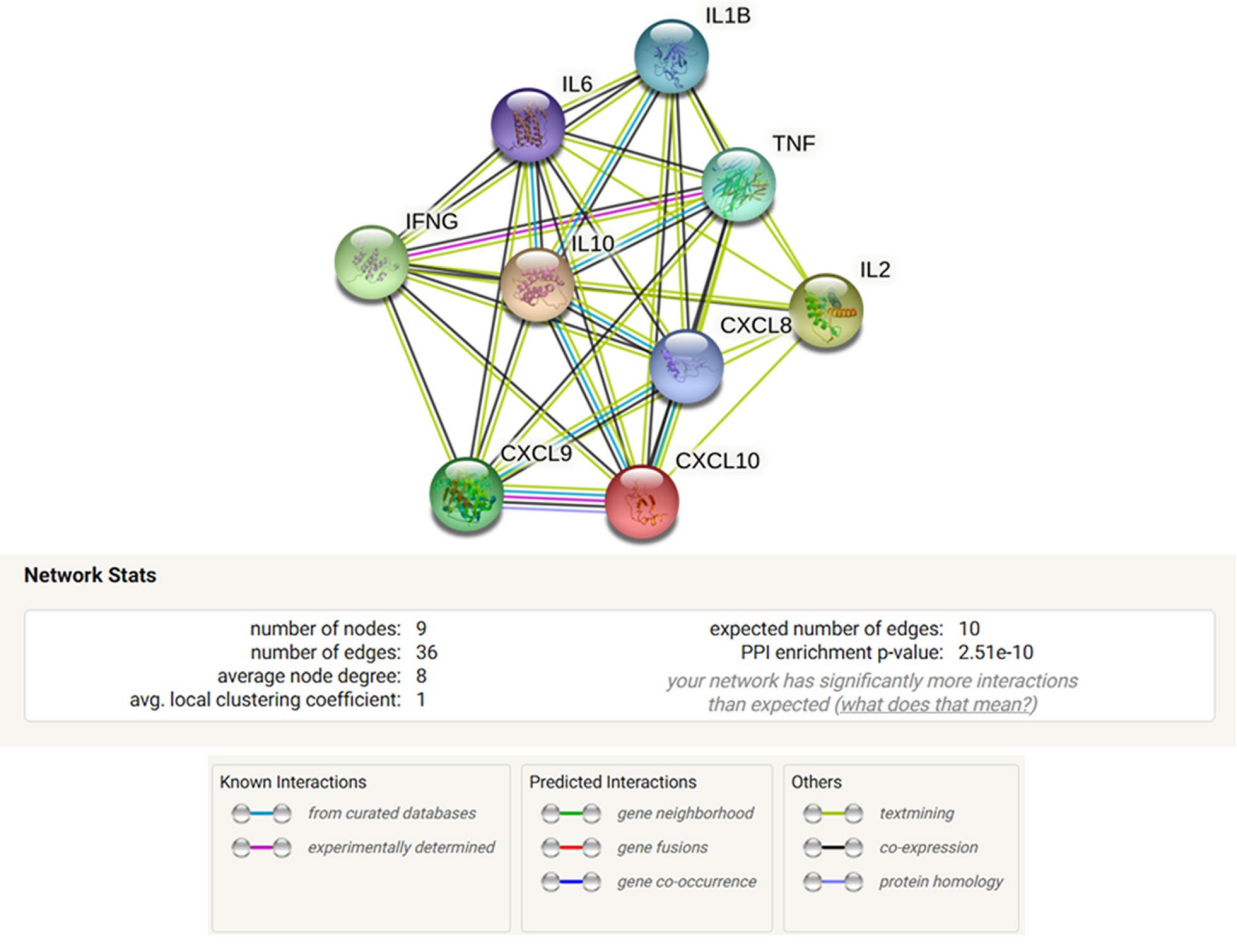
The above figure shows the protein–protein interactions and its network statistics of the significantly altered cytokines in the current study, among chronic SJS patients when compared with normal and the cytokines involved in specific biological processes are detailed in Table 3.

The co-expression analysis between 18 analytes revealed the RNA co-expression patterns, and protein co-regulation in the homeostatic conditions (Fig. 3). The scores of CXCL9 and CXCL10 were observed to be 0.848, which was the highest among all the analytes. IL-6 and CXCL8/IL-8 showed the second highest RNA co-expression score of 0.581. String analysis revealed the protein–protein interactions between nine selected proteins i.e., IL-1β, IL-2, IL-6, CXCL8/IL-8, IL-10, TNF-α, IFN-γ, CXCL9, CXCL10 (Fig. 4). The network statistics were 9 number of nodes, 36 number of edges and PPI enrichment value was 2.51e−10. Various biological processes are found to be involved in the development of chronic SJS which includes nitric oxide biosynthesis, regulation of apoptotic processes, response to chronic inflammation, defence response and the cellular response to the drug. The cytokines involved in the particular process has been tabulated for better understanding (Table 3).

**Table 3:** This table illustrates the significantly altered cytokines and the biological processes which correlates with the clinical ocular condition of chronic SJS patients.

GO term	Biological process	Cytokines involved	*p* value
GO:0002874	Regulation of chronic inflammatory response to antigenic stimulus	IL-10, TNF	1.55e−05
GO:0045428	Regulation of nitric oxide biosynthetic process	IFNG, IL-1B, 10, TNF	1.97e−07
GO:0035690	Cellular response to drug	IL-1B, 6, TNF	0.0011
GO:0045765	Regulation of angiogenesis	IL-1B, 6, 8, 10, CXCL10	1.00e−06
GO:0050900	Leukocyte migration	IL-1B, 6, 8, 10, TNF, CXCL9, CXCL10	1.46e−09
GO:0006952	Defense response	IL-1B, 6, 8, 10, IFNG, TNF, CXCL9, CXCL10	7.65e−08
GO:0022603	Regulation of anatomical structure morphogenesis	IL-1B, 6, 8, 10, TNF, CXCL9, CXCL10	4.11e−07
GO:0042981	Regulation of apoptotic process	IL-1B, 2, 6, 10, TNF, IFNG	8.62e−05

### Comparison of cytokine studies in chronic SJS tears

Till now, there are three reports from distinct groups that studied the tear cytokine levels in chronic SJS; one study from India, and other two from Japan. These studies have analysed different cytokine levels in 50–155 eyes. Gurumurthy et al. reported the significantly altered cytokines involved in chronic SJS tears, in response to lid margin keratinization, among pre and post mucous membrane grafting (MMG)^12^. Ueta et al. had compared the cytokines between atopic keratoconjunctivitis and chronic SJS^13^ and recently the same group (Yoshikawa et al.) had updated that CXCL8/IL-8 and IP-10 was involved in conjunctivalization and neovascularization respectively, while GrzB has role in keratinization by correlating with the clinical parameters from Ocular surface grading in chronic SJS patients^14^. The comparative analysis among the four studies including this had revealed that CXCL8/IL-8 and CXCL10 are commonly identified among inflammatory factors in all the studies (Table 4). Similarly, CXCL8/IL-8 levels were shown to be upregulated and IP-10/CXCL-10 were downregulated in all the four studies. These can be considered to be the common signature cytokine pattern in Chronic SJS ocular surface.

**Table 4:** Summary of differentially regulated inflammatory factors (pro-inflammatory and anti-inflammatory, ELR-negative CXC chemokines) comparing with severe DED and other previously existing studies among Stevens Johnson syndrome compared with age and gender matched controls of respective studies.

Significantly altered analytes	Our study		Significance of analytes in SJS compared with healthy controls		
	Comparision with healthy controls	Comparision with severe DED	Gurumurthy et al.	Ueta et al.	Yoshikawa et al.
**Pro inflammatory factors**					
IL-1a	–	–	–	–	↑
IL-1b	↑	ns	↑↑↑	–	–
IL-2	↑	ns	↑↑	–	–
IL-6	↑↑	↑	–	↑↑↑	↑
CXCL8/IL-8	↑↑	ns	↑↑	↑↑	↑↑
IL-15	ns	ns	↑	–	–
IL-17A	ns	ns	↑↑↑	–	–
TNF-alpha	ns	ns	↓↓↓	–	ns
IFN- γ	ns	ns	↓↓↓	↑↑	ns
Bfgf	ns	ns	↑↑↑	–	ns
RANTES/CCL5	ns	ns	↓↓	↑	ns
MCP-1/CCL2	ns	ns	↑↑↑	ns	ns
GMCSF	ns	↓↓↓	↑↑↑	–	–
GranzymeB	–	–	–	–	↑
Eotaxin				↑↑	↑
MIP-1b/CCL4	ns	ns		↑↑	↑
CD178	–	–	–	–	ns
VEGF	–	–	↓↓↓	–	–
IL-12p70	–	–	↓↓↓	–	–
IL-7	–	–	↓↓↓	–	–
**Anti inflammatory factors**					
IL-10	ns	↓	↓↓↓	–	–
IL-13	–	–	↓↓↓	–	ns
**ELR-negative CXC chemokines**					
CXCL10/IP-10	↓	↓↓	↓↓↓	↓↓	↓
CXCL9	ns	ns	–	–	–

*ns* non-significant, ‘–’ is not studied.

↑—p value ≤ 0.05, ↑↑—p value ≤ 0.01, ↑↑↑—p value ≤ 0.001.

## Discussion

This study tried to explore and discern the differential expression of various pro- and anti-inflammatory cytokines and chemokines in the tear film and ocular surface of patients with chronic ocular complications of SJS. We compared our findings with age and gender-matched healthy controls and patients with aqueous deficiency DED. Additionally, we compared our findings with the results of other similar studies in which the tear cytokine profile in chronic SJS has been reported (Table 3). The probable reasons for the differential regulation and their possible inter-relationship in the development of ocular disease in SJS have been unravelled but needs further validation in future studies.

Stevens–Johnson syndrome is an immune-mediated disorder, in the chronic sequelae, ocular surface is destroyed (as shown in Fig. 1) due to the damage that occurred in the acute stage^15,16^. Even though there are various inflammatory cytokines reported to understand the etio-pathogenesis of SJS, still there is a lack in identifying the signature inflammatory factor for the diagnosis and treatment of SJS. The common biological pathways and its interaction with the inflammatory factors need to be established to uncover the pathogenesis of SJS. Our study shows similar trends with respect to expression pattern of inflammatory cytokines studied previously in chronic SJS patients but differ with the level of significance of IL-10, IL-15, IL-17A, Bfgf, RANTES/CCL5, MCP-1, GMCSF, MIP1B/CCL4, TNF-α, and IFN-γ^12^. Also, we couldn’t find any significant difference among pre-MMG and post-MMG as reported in Gurumurthy et al., because of the modest sample size (3 post MMG patients). The reason behind selecting CXCL9 along with CXCL10 among ELR-negative CXC chemokines is because of their co-expression and similar intracellular domains for the activation of CXCR3^17^. And to check if they are co-expressing in chronic SJS ocular surface or not, but interestingly they are not co-expressive. CXCL10 was significantly downregulated and CXCL9 doesn’t exhibit any difference in chronic SJS tears when compared to normal and patients with severe DED.

In the current study we also used String, a bioinformatic tool for analysis of the significantly altered cytokines belonging to pro-inflammatory cytokines, anti-inflammatory cytokines, and ELR-negative CXC chemokines. The significantly altered cytokines in chronic SJS with healthy controls and severe DED were selected for understanding the role of each cytokine in biological pathways, the String analysis was utilized. The String analysis of significantly altered cytokines has shown that TNF-α and IL-10 are involved in the chronic inflammatory response in chronic SJS tears. It has been previously reported that nitric oxide levels were significantly increased in the serum of acute SJS patients^18^. This study has been helpful in understanding that, among the differentially regulated cytokines in chronic SJS, IFN-γ, IL-1β and TNF-α were significantly involved in the regulation of nitric oxide biosynthesis. However, the String analysis has shown that IL-1β, 6, 8, 10 and CXCL10 were involved in the biological process in regulation of angiogenesis. We still need to consider the limitation of all the data base on which String analysis do prediction using inbuilt algorithms and it aims to track only all available protein association.

It has been reported earlier that CXC chemokines that contains the ELR motif are potent promoters of angiogenesis and those that lack ELR are potent inhibitors of angiogenesis^19^. This difference in angiogenic activity may impact the pathogenesis of variety of disorders. CXCL8/IL-8 is an ELR motif-containing cytokine, which is highly expressing in the ocular surface of chronic SJS can be the promoter of angiogenesis in SJS. In the same way CXCL10, ELR-negative chemokine is an inhibitor of angiogenesis is down regulated in SJS. Simultaneously, the imbalanced expression of CXCL8/IL-8 and CXCL10 were also observed in other fibro-proliferative disorders i.e., Idiopathic pulmonary fibrosis (IPF)^20^. The exogenous IP‐10 (intramuscular) was administered in the bleomycin‐induced pulmonary fibrosis murine model, which resulted in marked attenuation of pulmonary fibrosis that was entirely attributable to a reduction in angiogenesis in the lung^21^.

On the flip side, among the co-expression of these analytes, CXCL8/IL-8 and IL-6 were found to be co-expressive and are capable of highly expressing pro-inflammatory cytokines in SJS tears. While CXCL10 and CXCL9 were highly co-expressive among all cytokines in our panel, but in SJS tears they were not consistent in terms of co-expression. We identified that CXCL10 levels are downregulated in the chronic SJS tear, unlike CXCL9 which has no significant difference in terms of expression even though they are highly co-expressive, in homeostatic conditions. The difference in the expression pattern of ELR-negative CXC chemokines in SJS might be because of the different effects of ligands on the variants of its receptors^17^. Also, these ligands have different temporal and spatial expressions in distinct cell types. However, these three ligands activate the same receptor but exhibit unique and differential expression which has been reported in various diseases like psoriasis^22^.

The main limitations of our study were the modest sample size and inability to correlate the ocular cytokine expression with the systemic level inflammatory factors. Since SJS is an extremely rare disease, the limitation in sample size is expected. Additionally, Out of 12 patients in the study, 11 had similar grading for eyelid, conjunctival, and corneal findings in both eyes, thus reflecting similarity in the magnitude of chronic ocular complications bilaterally (Table 2). Since most patients had severe dry eye as a component of the severe ocular complications, we did not get enough yield for the tear samples, and hence had to pool the tear samples from both eyes of the same patient. Therefore, we were unable to study the potential variability between eyes which might provide better insight of disease pathophysiology.

However, the study also has significant strengths including the comparison with similar ocular surface inflammatory condition i.e., severe DED tears, String analysis and a literature review to compare the expression profiles across different studies.

In conclusion, this study investigated the differential expression of tear cytokine levels among chronic SJS patients to understand the role of significantly altered cytokines in disease development. The study found that there was a differential expression of tear cytokines in SJS when compared to DED and healthy controls. The differential expression of CXCL8/IL-8 and CXCL10 was in line with existing literature and the role of these specific cytokines in chronic SJS pathogenesis merits further evaluation.

## Materials and methods

Tear samples were collected from 24 eyes (n = 12) of SJS/TEN patients, 36 eyes (n = 12) of age and gender-matched healthy controls, and 14 eyes (n = 7) of severe DED patients during the period of 2018 to 2019. The study protocol was approved by the institutional review board of LVPEI ethics committee and were performed under the tenets of the declaration of Helsinki. All the participants in this study had provided written informed consent before enrolling in this study. Written informed consent was obtained from all individuals for publication of images which were obtained from the electronic medical records. The demographics of participants of the study are detailed in the Table 1. The clinically diagnosed chronic SJS patients with a history of more than one year of onset of the reaction were recruited in this study. The diagnosis of SJS was confirmed based on the history of acute reaction in the skin and the mucous membrane involvement^23^. The criteria used to define severe dry eye was Schirmer score < 10 mm, OSDI score > 13 and positive corneal staining (> 1 on Oxford Staining Score (OSS))^24^.

### Tear collection and storage

The tear sample were collected from each eye of chronic SJS patients by performing Schirmer test 1 (without topical anesthesia) and the same has been followed in controls and severe dry eye disease (DED) patients. Initially Schirmer strip was placed in the cul-de-sac of the eye for 5 min (in case of controls, till it reaches 30 mm). These Schirmer strips with tears in it were placed under sterile conditions in a 500 µL punctured centrifuge tubes. The punctured 500 µL tubes were placed in 1.5 mL sterile centrifuge tubes as reported previously (Posa et al.)^25^ and samples were collected by centrifugation at 13,000 rpm for 5 min and immediately stored at − 80 °C until further use. The protein concentrations were estimated using the BCA method (Cat #786-570, G-Biosciences, Geno Technology Inc., USA).

### Estimation of tear cytokines using Luminex assay

The concentration of eighteen analytes were estimated using a customized panel of Luminex ELISA (LXSAHM-18, USA R&D Systems, Inc). The list of analytes measured were CCL2/JE/MCP-1, CCL4/MIP-1 beta, CCL5/RANTES, CCL11/Eotaxin, CXCL9/MIG, CXCL10/Interferon-γ induced protein-10/CRG-2, Fibroblast Growth Factor-basic/FGF2/bFGF, Granulocyte Monocyte-Colony Stimulating Factor, Interferon-γ, IL-1 β/IL-1F2, IL-2, IL-6, CXCL8/IL-8, IL-10, Interleukin-13, Interleukin-15, Interleukin-17 and tumour necrosis factor-α. All the reagents were allowed to obtain to room temperature (RT) prior to the experiments. The reagents were prepared according to the manufacturer’s guidelines. The total sample volume was adjusted to 35 µL with 150 mg/mL of total protein concentration. After the normalization of samples and preparation of standards, 50 µL of standards and 35 µL of the sample was added in appropriate wells as labelled. To these wells, 50 µL of diluted microparticle cocktail consisting of 18 analytes were added and followed by incubation for 2 h at room temperature on a shaker at 800 rpm. After incubation, the wells were washed thrice with 100 µL of wash buffer followed by incubation with 50 µL of diluted biotin-antibody cocktail for 1 h at RT on the shaker at 800 rpm. After incubation, 50 µL of diluted streptavidin-PE was added to each well and incubated for 30 min at room temperature at 800 rpm. Finally, the assay plate was decanted and washed 3 times with 100 µL of wash buffer. In the end, 150 µL of wash buffer was added and incubated for 2 min at RT on the shaker at 800 rpm and then read on a Luminex reader by using XPONENT 4.2 software using default parameters and results were obtained in Median fluorescent intensity (MFI), which were further used to analyse the data.

### String analysis

The String (Search Tool for Retrieval of Interacting Genes/Proteins) biological database (version 11), was used to perform the co-expression analysis for better understanding of the RNA co-expression patterns and protein co-regulation in SJS patients. The network type was indicating the edges with both functional and physical protein associations. Default prediction methods were used with high confidence scores at > 0.7. The analytes given to the String database were gene names of all the analytes in our customized panel i.e., IL1β, CCL2, CCL4, CCL5, CCL11, CXCL9, CXCL10, FGF, GMCSF, IFN-γ, IL2, IL6, IL8/CXCL8, IL10, IL13, IL15, IL17 and TNF-α. The heat map was generated using the software, giving the RNA co-expression patterns, and protein co-regulation provided by ProteomeHD in String database.

The protein–protein interactions were established for the significantly altered inflammatory cytokines IL6, IL1β, IL8, IL10, IL2, two anti-fibrotic cytokines TNF-α, IFN-γ and ELR-negative CXC chemokines i.e., CXCL10 and CXCL9. The analysis of this protein–protein interaction network was used for better understanding of the roles of cytokine in a biological processes.

### Comparison of cytokine studies in chronic SJS tears

To correlate the cytokine studies reported till now in Chronic SJS tears, we have done the PubMed search with the keywords, Cytokine/SJS/Chronic. PubMed displayed 21 search reports for the given keywords. Out of these, 3 studies were found to be similar to compare with our study in terms of sample type (tear), chronic stage patients, age group (adult) of the patient and the duration from onset of disease. All the analytes studied till now as mentioned in the above three studies are correlated with our study and has been tabulated. The significance has been compared between the selected studies for the cytokines in SJS and the healthy controls of the respective studies.

### Statistical analysis

Statistical analysis was performed using GraphPad Prism 5 in between the three groups SJS, Control, and severe DED. Data was expressed as the mean and evaluated with Student’s t-test. Graphical representation has been done by using whiskers Tukey. p-value ≤ 0.05 was considered as significant and ≤ 0.001 as highly significant.

## Supplementary Information

Supplementary Information.
