# Supplementary material for: Differential expression of tear film cytokines in Stevens–Johnson syndrome patients and comparative review of literature
Source: Sci Rep. 2021 Sep 16;11:18433. doi: 10.1038/s41598-021-97575-y (PMC8446064; doi:10.1038/s41598-021-97575-y)
Supplement: Supplementary file 1 — Supplementary Information. [file 41598_2021_97575_MOESM1_ESM.docx]

| Patient | Topical steroid/anti inflammatory drug used  (both eyes) | Dosage |
| --- | --- | --- |
| Patient 1 | Ciclosporin | 0.05% w/v |
| Patient 2 | Tacrolimus | 0.03% w/w |
| Patient 3 | Tacrolimus | 0.03% w/w |
| Patient 4 | NA | NA |
| Patient 5 | Tacrolimus | 0.03% w/w |
| Patient 6 | NA | NA |
| Patient 7 | NA | NA |
| Patient 8 | Tacrolimus | 0.03% w/w |
| Patient 9 | NA | NA |
| Patient 10 | Fluorometholone | 0.1% w/v |
| Patient 11 | NA | NA |
| Patient 12 | NA | NA |

Supplementary table 1: The table indicates the topical steroid/anti inflammatory drug used in the patients with the respective dosage. NA= Not on topical steroids/anti-inflammatory usage.
